# Inflammation mediation of the association between brominated flame retardants and psoriasis among U.S. adults

**DOI:** 10.3389/fpubh.2025.1602943

**Published:** 2025-12-10

**Authors:** Ming Jiang, Lifan Jiang, Wanqian Li, Jiahui Zhang, Sujun Tang, Ying Peng, Xia Zhang, Yi Zhang, Kesu Hu

**Affiliations:** 1 Department of Burn and Plastic Surgery, Affiliated Hospital of Nantong University, Nantong, Jiangsu, China; 2 Nantong University Medical School, Nantong, Jiangsu, China

**Keywords:** psoriasis, brominated flame retardants, inflammation, NHANES, mediation

## Abstract

**Background:**

Brominated flame retardants (BFRs), widely applied in fire prevention, are persistent environmental pollutants. Accumulating evidence indicates that BFRs can trigger inflammatory responses in humans, potentially contributing to various diseases. Given the central role of inflammation in psoriasis pathogenesis, investigating the association between BFR exposure and psoriasis risk is of considerable importance.

**Methods:**

We analyzed data from the 2005–2006 and 2009–2014 cycles of the National Health and Nutrition Examination Survey (NHANES). concentrations of BFRs were natural logarithmic transformed and categorized into quartiles. The association between BFR exposure and psoriasis was evaluated using multivariable regression models, restricted cubic spline analysis, mediation analysis, and interaction testing to assess both individual and combined effects.

**Results:**

Among 4,251 adult participants, 123 (2.9%) reported having psoriasis. After adjusting for covariates, individuals in the highest quartile of polybrominated diphenyl ether 153 (PBDE153) exposure (Q4) exhibited a significantly increased risk of psoriasis compared to those in the lowest quartile (Q1) (OR = 2.89, 95% CI: 1.26–6.61, *p* = 0.013). Restricted cubic spline analysis indicated a positive dose–response relationship between PBDE153 levels and psoriasis risk (P-overall = 0.012, P-nonlinear = 0.881), with a significant linear trend (*p* < 0.001). Weighted Quantile Sum (WQS) analysis revealed a statistically significant positive association between the BFR mixture and psoriasis risk (OR = 1.709, 95% CI: 1.241–2.370, *p* = 0.001). PBDE153 was identified as the primary contributor, with a weight of 0.691. In contrast, Quantile G-computation (Qgcomp) analysis showed a positive but non-significant association (psi1 = 0.273, 95% CI: −0.043 to 0.589, *p* = 0.090). Notably, individuals with higher poverty income ratios exhibited increased susceptibility to psoriasis risk associated with PBDE153 (*p* = 0.016). Inflammation accounted for 4.35% of the relationship between PBDE153 and psoriasis (*p* < 0.05).

**Conclusion:**

This study suggests a positive association between PBDE153 exposure and psoriasis risk, with inflammatory pathways playing a partial mediating role. However, the cross-sectional design precludes causal inference. Therefore, longitudinal studies and experimental research are needed to establish such causal relationships.

## 1 Introduction

Brominated flame retardants (BFRs) possess exceptional flame-retardant properties and are widely incorporated into a variety of consumer products, including plastics, furniture, upholstery, electronic devices, textiles, and household items (1–3). These compounds readily enter the environment through volatilization and seepage due to their weak chemical bonds with product materials. As a result, BFRs have become ubiquitous environmental contaminants, impacting atmospheric, aquatic, sedimentary, and terrestrial ecosystems (4). BFRs are lipophilic and can accumulate in adipose tissues, with exposure occurring primarily through diet, inhalation, and dermal contact (5). These compounds also exhibit bioaccumulation and biomagnification properties (6, 7). Previous research has established that certain BFRs can activate bone marrow-derived dendritic cells, which are crucial components of the immune system. *In vitro* studies indicate that BFR exposure enhances the expression of MHC class II, CD86, and T-cell receptors (TCR) in splenocytes, as well as elevates interleukin (IL)-4 production (8). Furthermore, BFRs have been linked to endocrine disruption, reproductive issues, liver dysfunction, and neurobehavioral alterations (9, 10). Despite efforts to phase out BFRs, they continue to be detected in consumer products, food items, household dust, and indoor environments (11).

Given that dermal contact is a primary route for the absorption of BFRs and that these compounds are lipophilic, it is plausible that the accumulation of BFRs in the skin and subcutaneous tissues may contribute to certain skin disorders. Supporting this hypothesis, there are notable parallels between the potential effects of BFRs and the etiological mechanisms underlying psoriasis, both of which involve inflammatory processes. Psoriasis manifests as accelerated keratinocyte proliferation, resulting in erythematous, scaly plaques. This process is thought to be orchestrated by various cytokines, notably IL-23, IL-17, and tumor necrosis factor-alpha (TNF-α), which drive inflammation and characteristic symptoms (12, 13). Psoriasis is not merely a dermatological condition but a systemic disorder that can significantly impact the joints and internal organs, leading to comorbidities such as psoriatic arthritis, cardiovascular diseases, and metabolic syndrome. Additionally, psoriasis negatively affects quality of life, causing psychological distress, social isolation, and reduced productivity (14). Globally, the prevalence of psoriasis is estimated to affect approximately 2–3% of the population (15). In Ontario, Canada, this prevalence increased from 1.74% in 2000 to 2.32% in 2015, reflecting heightened awareness and the growing impact of the condition on public health (16). Furthermore, the economic burden of psoriasis is substantial, with direct medical costs in the United States exceeding $5 billion annually (17). Despite advances in treatment, psoriasis remains incurable, underscoring the importance of identifying environmental factors to aid in its prevention (18).

As a class of persistent organic pollutants, the potential connection between BFR exposure and the pathogenesis of psoriasis has not yet been thoroughly investigated. Our study analyzes data from the National Health and Nutrition Examination Survey (NHANES) covering the years 2005–2006 and 2009–2014 to clarify the potential relationship between BFR exposure and the incidence of psoriasis in humans.

## 2 Methods

### 2.1 Study population

Data were sourced from the NHANES, a comprehensive cross-sectional study conducted by the Centers for Disease Control and Prevention (CDC) and the National Center for Health Statistics (NCHS) to evaluate the nutritional and health status of the U.S. population. The study protocol received approval from the NCHS Research Ethics Review Board, and all participants provided written informed consent.

**Figure 1:**
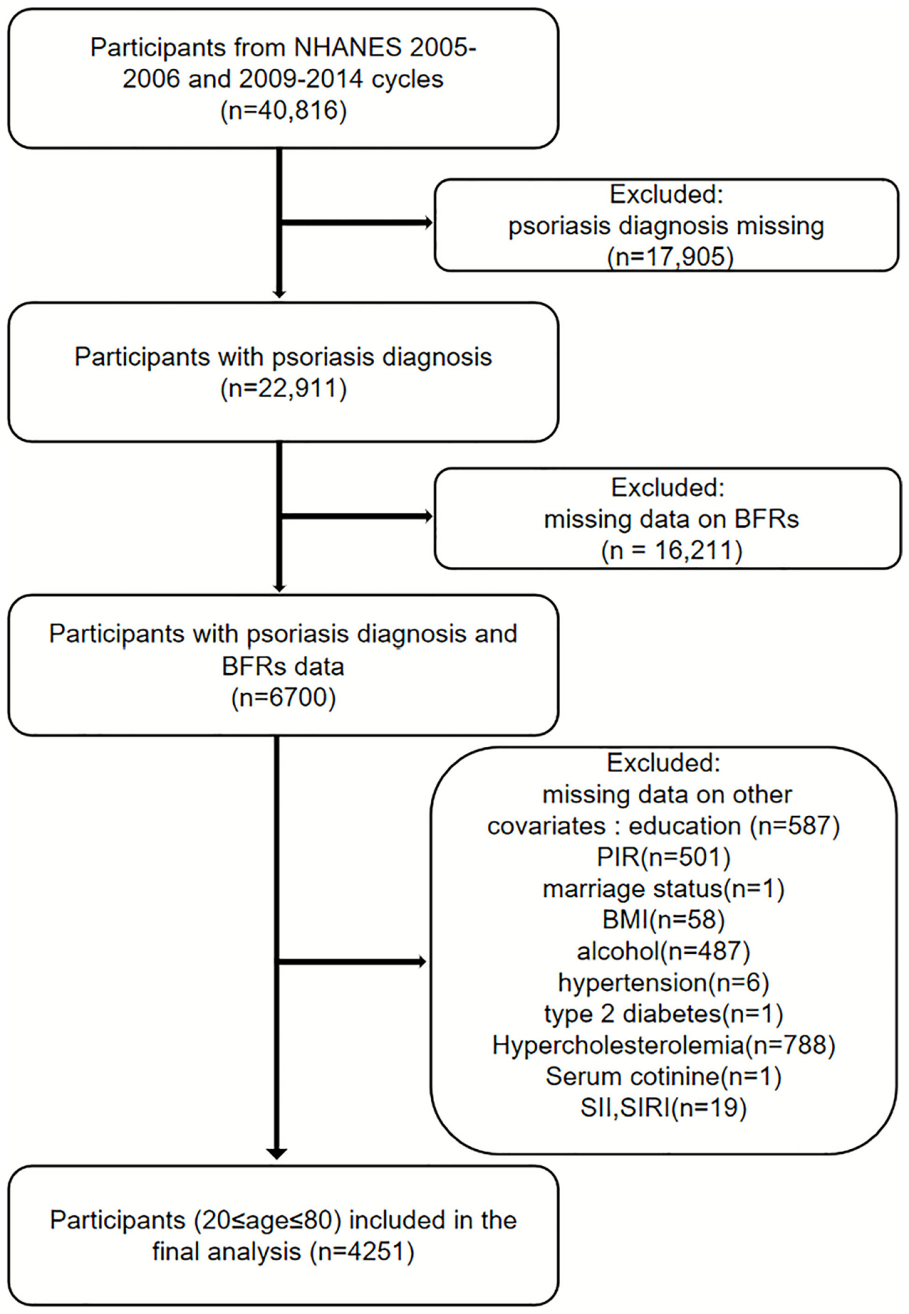
The flow diagram.

Serum BFR measurements were available for 6,700 participants across four NHANES cycles (2005–2006 and 2009–2014). After excluding participants with incomplete data on BFRs, psoriasis, and other covariates, the final analytical sample consisted of 4,251 participants (Figure 1).

### 2.2 Brominated flame retardants assessment

Serum BFR concentrations were determined using automated liquid/liquid extraction and purification methods, followed by quantification via Isotope Dilution High-Resolution Gas Chromatography–Mass Spectrometry (GC/MS) (19). Given that organohalogen compounds are lipophilic and distributed in the body according to tissue lipid content, lipid-adjusted concentrations were used for all analyses. This approach better reflects the levels of these chemicals in adipose tissue and reduces variability by accounting for individual differences in serum lipid levels (20). Consequently, all chemical concentrations in this study are expressed as ng/g lipid.

For measurements below detection thresholds, values were standardized by dividing the detection limit by the square root of two. Following established research protocols, we focused on nine BFRs with detection rates exceeding 70% to ensure robust analytical outcomes (21). The selected compounds and the percentages of samples below the LOD are presented in Supplementary Table S1.

### 2.3 Psoriasis

Participants were classified as having psoriasis if they responded affirmatively to either of the following inquiries: “Have you ever been told by a doctor or another healthcare professional that you have psoriasis?” or “Have you ever been told by a healthcare provider that you have psoriasis?” Individuals who provided uncertain responses or declined to answer were excluded from our analysis (22).

### 2.4 Covariates

Potential confounding factors associated with psoriasis were identified based on prior research. These covariates included gender (female, male), age (20–65 years, >65 years), race/ethnicity (Mexican American, other Hispanic, non-Hispanic Black, non-Hispanic White, other race), education level (less than 9th grade, 9th–11th grade, high school graduate, some college or associate degree, college graduate or above), family poverty income ratio (PIR: <5, ≥5), marital status (never married, married, separated, divorced, living with partner, widowed), body mass index (BMI: <25 kg/m^2^, 25–29.9 kg/m^2^, ≥30 kg/m^2^), alcohol intake (≥12 times/year, <12 times/year), serum cotinine levels (ng/mL), and the presence of comorbid conditions such as hypertension, diabetes, and hypercholesterolemia (no/yes for each condition). Systemic inflammatory markers were also evaluated, including the systemic inflammation response index (SIRI) and the systemic immune-inflammation index (SII), which were calculated using the following formulas: SIRI = (monocyte count × neutrophil count)/lymphocyte count; SII = (platelet count × neutrophil count)/lymphocyte count (22). Participants’ baseline status for hypertension, hypercholesterolemia, and type 2 diabetes was determined through physician diagnosis (23, 24), with each condition classified as either present or absent.

### 2.5 Statistical analysis

All analyses were conducted using IBM SPSS Statistics 27 and R (version 3.6.3). We pooled data from four NHANES cycles using the unique respondent sequence number (SEQN). As per the recommendations of the National Center for Health Statistics (NCHS), a new survey weight was created for the combined dataset by dividing the original 2-year MEC examination weight (WTmEC2YR) by the number of cycles. To account for the complex survey design (including clustering and stratification), all primary analyses incorporated this adjusted weight, along with the stratification (SDMVSTRA) and primary sampling unit (SDMVPSU) variables, using the svydesign function in the R survey package. For the mediation analysis conducted in SPSS, the survey weight was applied using the case weighting function to ensure population representativeness.

Between-group comparisons of participant characteristics based on psoriasis status utilized Wilcoxon rank-sum tests for continuous variables and chi-square tests for categorical variables. To achieve a normal distribution, the concentrations of polychlorinated biphenyl 153 (PBB-153) and polybrominated diphenyl ethers (PBDEs) underwent natural logarithmic transformation. These values were categorized into quartiles (Q1–Q4) for categorical analyses. The relationship between individual BFRs and psoriasis was examined using both univariate and multivariate logistic regression models. We conducted diagnostic checks to assess the influence of individual observations on our regression results. Cook’s distance was calculated for the multivariable logistic regression model to identify influential observations that might disproportionately affect the parameter estimates. Following established guidelines in regression diagnostics (25, 26), we used a conservative threshold of Cook’s distance >1 to define influential observations. A sensitivity analysis was then performed by excluding these influential points to evaluate the robustness of our primary findings. Potential multicollinearity among BFRs was evaluated using Pearson correlation coefficients and visualized through heatmap analysis. To assess the cumulative impact of multiple BFRs, we employed two complementary approaches: (1) Generalized Weighted Quantile Sum (gWQS) regression, which addresses challenges posed by correlated pollutants through a weighted quantile index. This analysis utilized a 40:60 training-validation split with 1,000 bootstrap iterations to ensure robust results (27). (2) Quantile g-Computation (Qgcomp) regression, which overcomes directional association constraints inherent in WQS regression (28). Dose–response relationships between BFR concentrations and psoriasis risk were modeled using restricted cubic splines (RCS) and visualized through curve plotting. To account for multiple comparisons across the nine tested BFRs, false discovery rate (FDR) correction was applied using the Benjamini-Hochberg procedure. This correction was applied to *p*-values from the univariate and multivariate logistic regression models, the p for trend in the quartile analysis, and the overall *p*-value from the restricted cubic spline (RCS) analyses. The resulting *q*-values are reported alongside the original *p*-values, with *q* < 0.05 considered statistically significant. To address the high multicollinearity among PBDE congeners and to identify a parsimonious and most relevant set of risk factors, we performed a sensitivity analysis using the Least Absolute Shrinkage and Selection Operator (LASSO) regression. The multivariable LASSO model included the nine natural logarithm-transformed BFRs and all covariates (gender, age, race, education, PIR, marital status, BMI, alcohol intake, hypertension, diabetes, hypercholesterolemia, SII, SIRI, and serum cotinine), with psoriasis status (yes/no) as the outcome. The optimal penalty parameter (lambda) was determined by 10-fold cross-validation. We present results for both the model yielding the minimum cross-validation error (lambda.min) and the most regularized model with an error within one standard error of the minimum (lambda.1se). This analysis was implemented using the glmnet package in R. A parallel mediation framework was employed to investigate the potential mediating role of inflammation markers in the BFR-psoriasis relationship. This analysis was conducted using SPSS PROCESS macros with bootstrap-based significance testing (5,000 bootstrap samples). To optimize model parsimony and mitigate potential overadjustment, the covariates for the mediation model were selected through a data-driven approach using LASSO regression, retaining only variables with non-zero coefficients. The direct effect (DE) measured the unmediated impact of BFR exposure on psoriasis, while the indirect effect (IE) assessed its impact through a mediator. The mediation proportion was calculated as IE divided by the total effect (TE). Finally, subgroup analyses explored potential effect modifications through formal interaction testing.

## 3 Results

### 3.1 Baseline characteristics

Among the 4,251 participants from the NHANES cohorts (2005–2006 and 2009–2014), 123 (2.9%) reported having psoriasis (Table 1). The study population was well balanced in terms of gender (51.7% female, 48.3% male) and age distribution (50.9% ≤ 65 years, 49.1% > 65 years) between the two groups. The majority of participants were Non-Hispanic White (45.8%), had a PIR ≥ 5 (80.5%), were married (54.0%), and reported alcohol consumption ≥12 times/year (73.1%). Most participants did not have hypertension (62.2%), diabetes (84.0%), or hypercholesterolemia (62.1%). Notably, individuals with psoriasis exhibited significantly higher levels of SIRI and PBDE153 compared to those without psoriasis (both *p* = 0.001). A significantly greater proportion of participants aged >65 years was observed in the psoriasis group (59.3%) than in the non-psoriasis group (48.8%, *p* = 0.021).

**Table 1:** Baseline characteristics by psoriasis status.

Characteristics	Total	Without psoriasis	With psoriasis	*p*-value
*N* (%)	4,251	4,128 (97.1)	123 (2.9)	
Gender, *n* (%)				0.631
Female	2,199 (51.7)	2,138 (51.8)	61 (49.6)	
Male	2,052 (48.3)	1,990 (48.2)	62 (50.4)	
Age, *n* (%)				0.021*
<65	2,163 (50.9)	2,113 (51.2)	50 (40.7)	
≥65	2,088 (49.1)	2,015 (48.8)	73 (59.3)	
Race, *n* (%)				0.072
Mexican American	537 (12.6)	526 (12.7)	11 (8.9)	
Other Hispanic	387 (9.1)	374 (9.1)	13 (10.6)	
Non-Hispanic White	1,946 (45.8)	1,876 (45.4)	70 (56.9)	
Non-Hispanic Black	901 (21.2)	884 (21.4)	17 (13.8)	
Other race	480 (11.3)	468 (11.3)	12 (9.8)	
Education level, *n* (%)				0.556
Less Than 9th Grade	325 (7.6)	317 (7.7)	8 (6.5)	
9–11th Grade	567 (13.3)	549 (13.3)	18 (14.6)	
High School Grad	909 (21.4)	885 (21.4)	24 (19.5)	
Some College or AA degree	1,321 (31.1)	1,288 (31.2)	33 (26.8)	
College Graduate or above	1,129 (26.6)	1,089 (26.4)	40 (32.5)	
PIR, *n* (%)				0.998
<5	829 (19.5)	805 (19.5)	24 (19.5)	
≥5	3,422 (80.5)	3,323 (80.5)	99 (80.5)	
Marital status, *n* (%)				0.331
Married	2,295 (54.0)	2,239 (54.2)	56 (45.5)	
Widowed	329 (7.7)	321 (7.8)	8 (6.5)	
Divorced	466 (11)	447 (10.8)	19 (15.4)	
Separated	147 (3.5)	141 (3.4)	6 (4.9)	
Never married	717 (16.9)	693 (16.8)	24 (19.5)	
Living with partner	297 (7.0)	287 (7)	10 (8.1)	
BMI, *n* (%)				0.575
<25 kg/m^2^	1,151 (27.1)	1,122 (27.2)	29 (23.6)	
25–29.9 kg/m^2^	1,406 (33.1)	1,366 (33.1)	40 (32.5)	
≥30 kg/m^2^	1,694 (39.8)	1,640 (39.7)	54 (43.9)	
Alcohol intake, *n* (%)				0.665
<12 times/year	1,144 (26.9)	1,113 (27.0)	31 (25.2)	
≥12 times/year	3,107 (73.1)	3,015 (73.0)	92 (74.8)	
Hypertension, *n* (%)				0.222
No	2,643 (62.2)	2,573 (62.3)	70 (56.9)	
Yes	1,608 (37.8)	1,555 (37.7)	53 (43.1)	
Diabetes, *n* (%)				0.611
No	3,570 (84.0)	3,467 (84.0)	103 (83.7)	
Borderline	115 (2.7)	110 (2.7)	5 (4.1)	
Yes	566 (13.3)	551 (13.3)	15 (12.2)	
Hypercholesterolemia, *n* (%)				0.164
No	2,640 (62.1)	2,571 (62.3)	69 (56.1)	
Yes	1,161 (37.9)	1,557 (37.7)	54 (43.9)	
SII (10^3^ cells/mL)	460.82 (322.78,642.33)	459.51 (323.41,640.42)	497.00 (314.48,723.27)	0.251
SIRI (10^3^ cells/mL)	1.00 (0.69,1.47)	0.99 (0.68,1.45)	1.18 (0.82,1.77)	0.001**
Serum cotinine (ng/mL)	0.04 (0.01,2.36)	0.04 (0.01,2.41)	0.05 (0.01,0.94)	0.559
lnPBB153 (ng/g lipid)	2.17 (1.74,2.64)	2.17 (1.74,2.64)	2.09 (1.68,2.64)	0.729
lnPBDE28 (ng/g lipid)	0.20 (−0.18,0.54)	0.20 (−0.18,0.54)	0.17 (−0.17,0.54)	0.976
lnPBDE47 (ng/g lipid)	3.03 (2.66,3.47)	3.03 (2.66,3.47)	2.92 (2.68,3.45)	0.446
lnPBDE85 (ng/g lipid)	−0.92 (−1.35, −0.45)	−0.92 (−1.34, −0.45)	−1.05 (−1.46, −0.54)	0.124
lnPBDE99 (ng/g lipid)	1.37 (0.93,1.86)	1.37 (0.93,1.86)	1.29 (0.90,1.80)	0.262
lnPBDE100 (ng/g lipid)	1.41 (1.06,1.84)	1.41 (1.06,1.84)	1.39 (0.98,1.68)	0.070
lnPBDE153 (ng/g lipid)	0.99 (0.33,1.55)	0.98 (0.32,1.53)	1.16 (0.65,1.93)	0.001**
lnPBDE154 (ng/g lipid)	−0.96 (−1.37, −0.51)	−0.96 (−1.37, −0.51)	−1.13 (−1.52, −0.60)	0.082
lnPBDE209 (ng/g lipid)	0.98 (0.68,1.23)	0.98 (0.66,1.23)	0.99 (0.69,1.38)	0.570

The continuous variables were presented as median (interquartile range, IQR), and the categorical variables were presented as number (percentages). *N*, numbers of subjects; PIR, the family income to poverty ratio; BMI, body mass index; SII, systemic immune-inflammation index; SIRI, systemic inflammation response index; NHANES, National Health and Nutrition Examination Survey; In, natural logarithmic transformation. *p < 0.05, **p < 0.005.

**Figure 2:**
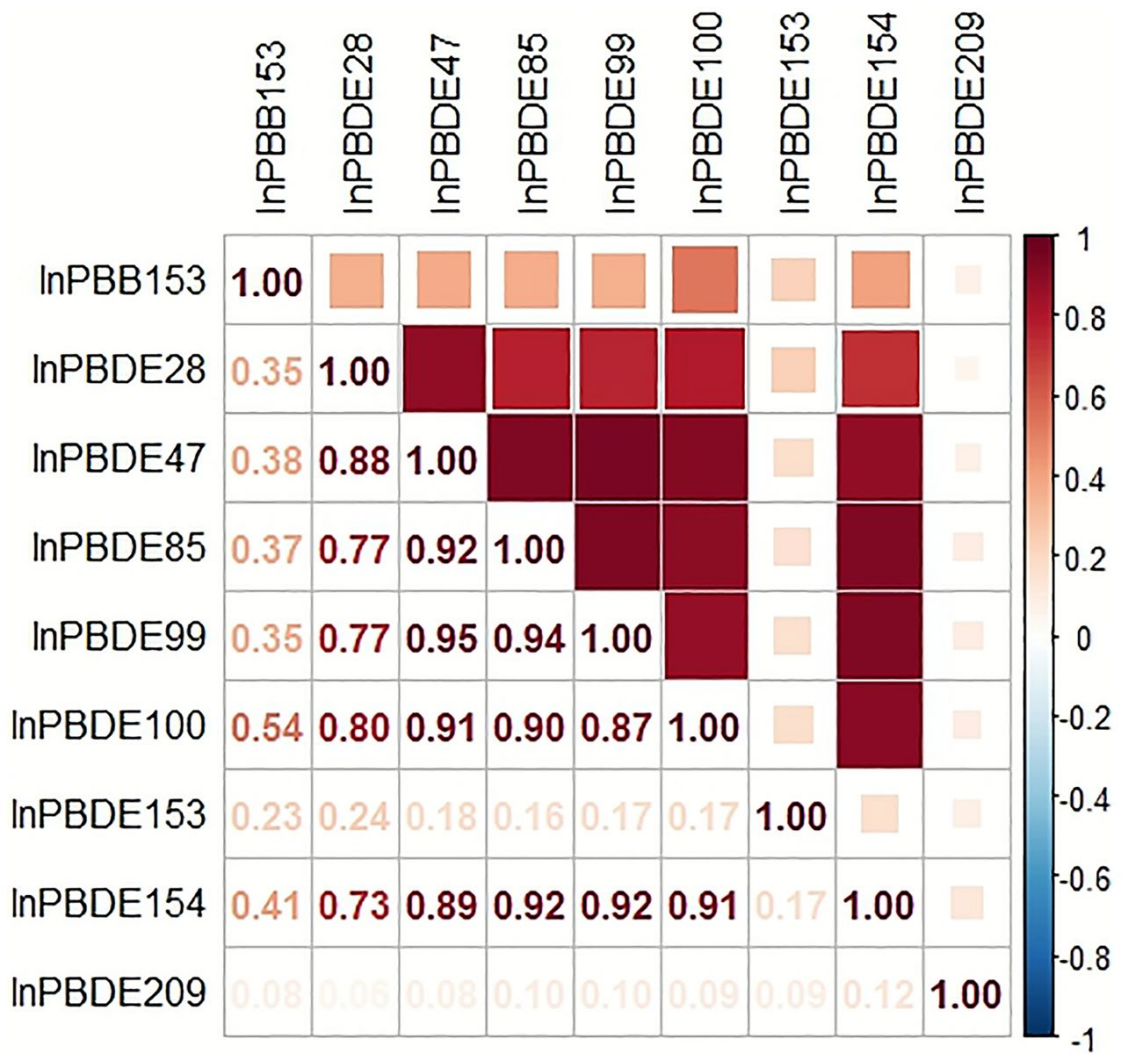
Correlations between BFRs.

Pearson correlation analysis of natural logarithm-transformed BFRs revealed a moderate correlation between PBB153 and PBDE100 (*r* = 0.58). In contrast, PBB153, PBDE153, and PBDE209 exhibited weak correlations with other BFRs (*r* ≤ 0.41). Strong correlations (*r* > 0.70) were observed among PBDE28, PBDE47, PBDE85, PBDE99, PBDE100, and PBDE154 (Figure 2).

### 3.2 Association between single BFR and psoriasis

**Figure 3:**
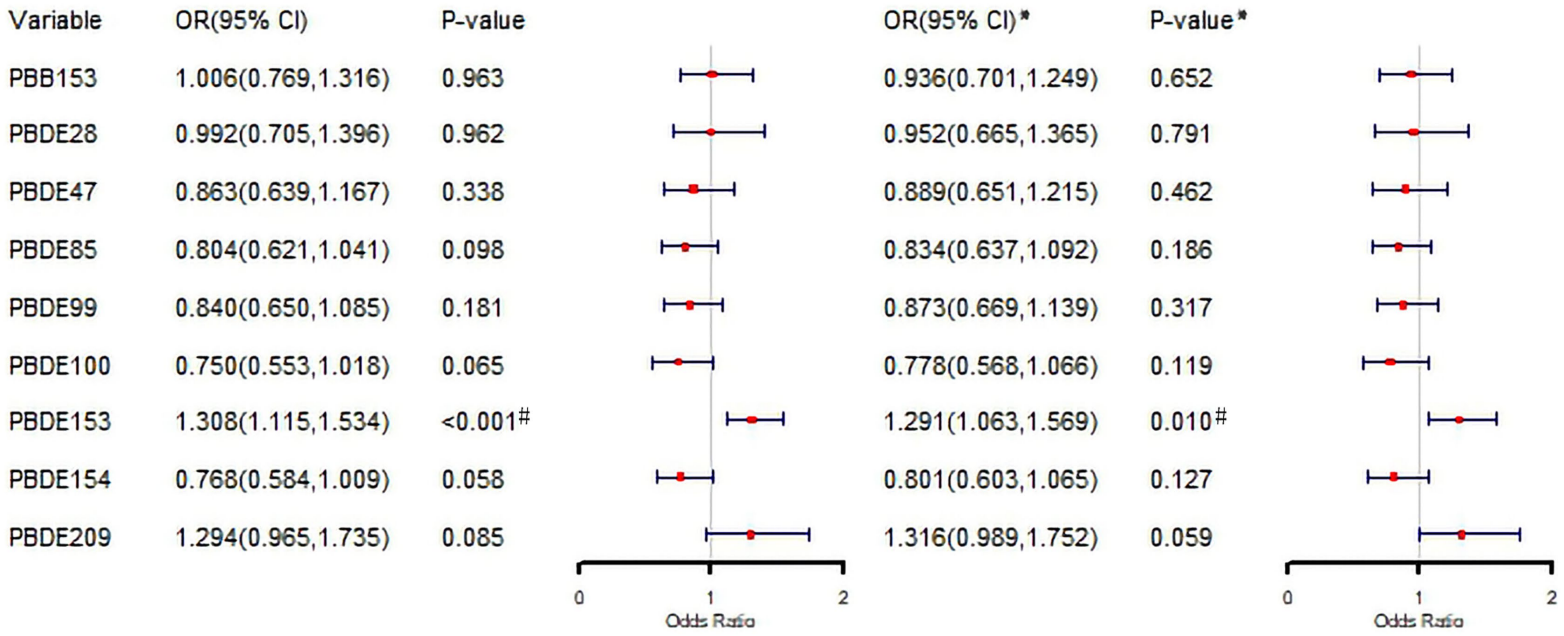
Associations between natural logarithm-transformed BFRs and psoriasis in logistic regression analysis. *Adjusted for gender, age, race, education level, PIR, marital status, BMI, alcohol intake, hypertension, diabetes, hypercholesterolemia, SII, SIRI, serum cotinine. OR, odd ratio; CI, confidence interval; PIR, the family income to poverty ratio; BMI, body mass index; SII, systemic immune-inflammation index; SIRI, systemic inflammation response index. ^#^*p* < 0.05.

Logistic regression analyses of natural logarithm-transformed BFRs revealed odds ratios (ORs) exceeding 1 for PBB153, PBDE153, and PBDE209, while other BFRs showed ORs below 1 (Figure 3). PBDE153 was the only compound to show a statistically significant association with psoriasis, with an unadjusted OR of 1.308 (95% CI: 1.115–1.534, *p* < 0.001) and an adjusted OR of 1.291 (95% CI: 1.063–1.569, *p* < 0.001) in the multivariable model. The model was adjusted for gender, age, race, education, PIR, marital status, BMI, alcohol intake, hypertension, diabetes, hypercholesterolemia, SII, SIRI, and serum cotinine.

**Figure 4:**
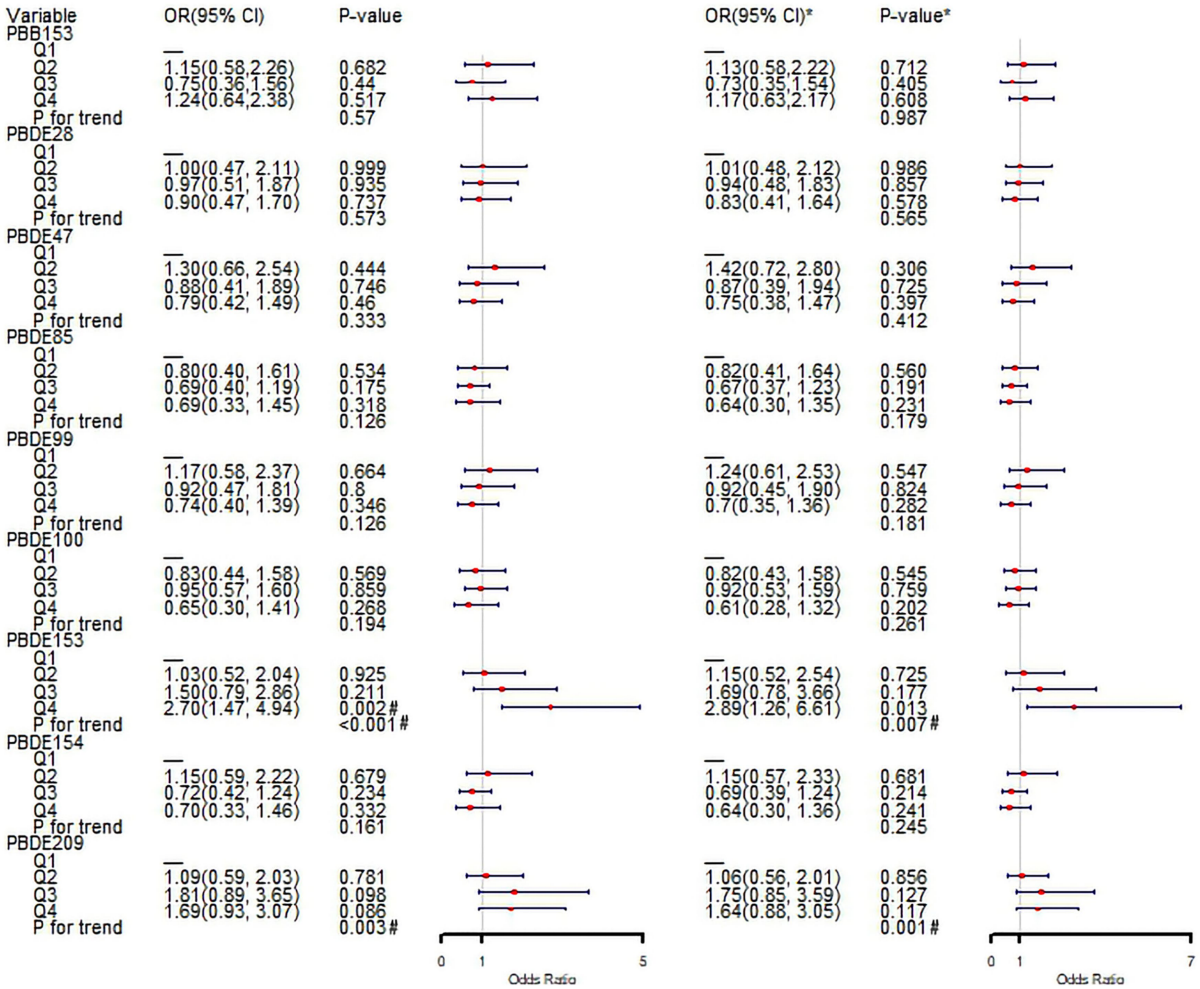
Associations between quartile groups of natural logarithm-transformed BFRs and psoriasis in logistic regression analysis. ^*^Adjusted for gender, age, race, education level, PIR, marital status, BMI, alcohol intake, hypertension, diabetes, hypercholesterolemia, SII, SIRI, serum cotinine. Q, quartile; OR, odd ratio; CI, confidence interval; PIR, the family income to poverty ratio; BMI, body mass index; SII, systemic immune-inflammation index; SIRI, systemic inflammation response index. ^#^*p* < 0.05.

Quartile analysis indicated that participants in the highest quartile of PBDE153 (Q4) had a significantly elevated risk of psoriasis compared to those in the lowest quartile (Q1), with an unadjusted OR of 2.70 (95% CI: 1.47–4.94, *p* = 0.002) and an adjusted OR of 2.89 (95% CI: 1.26–6.61, *p* = 0.013). Other quartile groups did not show significant risk differences compared to Q1. Linear trend analysis demonstrated an increasing risk of psoriasis across higher quantiles of PBDE153 and PBDE209, both before and after covariate adjustments (all P for trend < 0.01) (Figure 4).

**Figure 5:**
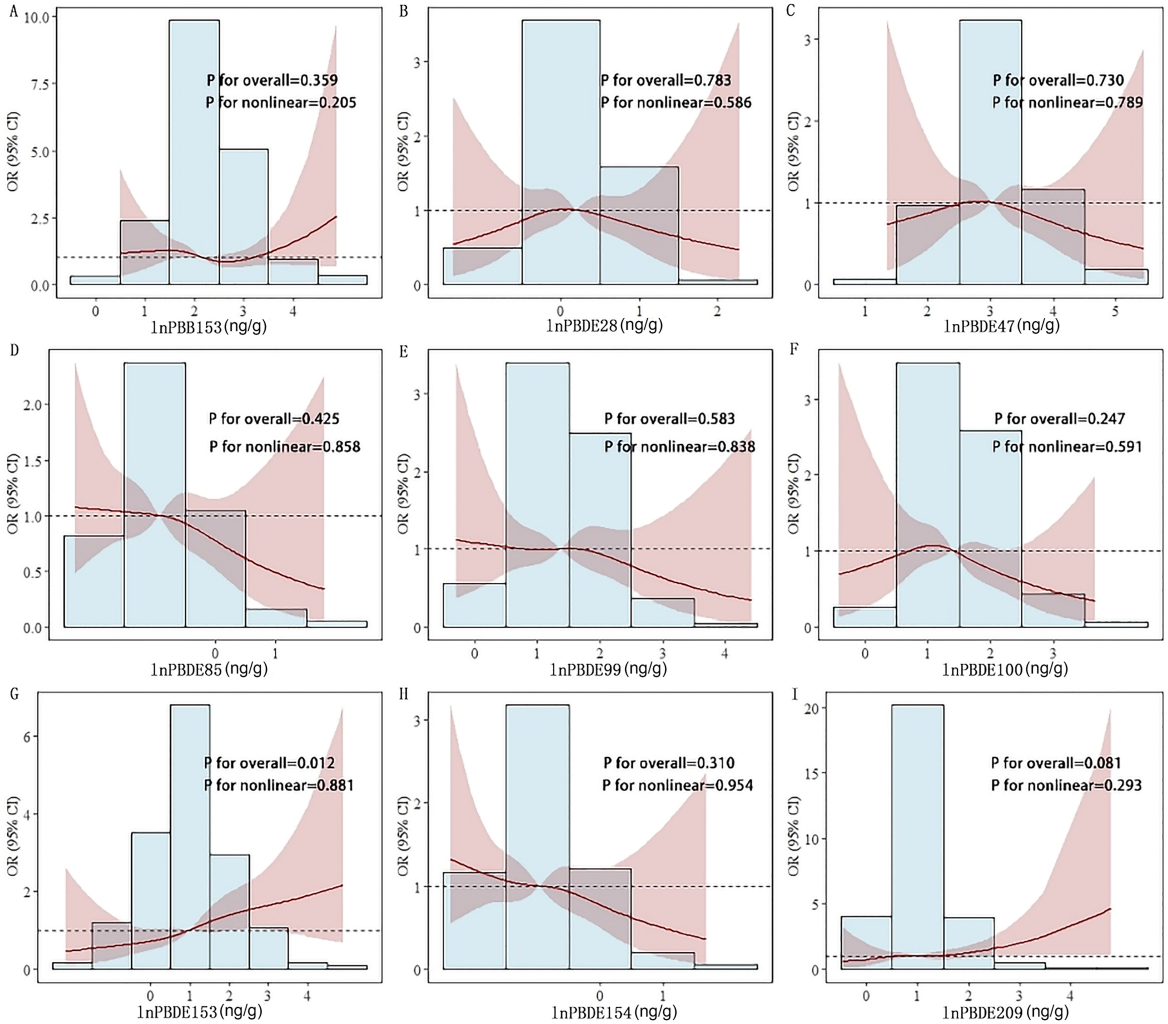
Restricted cubic spline fitting for the association between serum levels of polybrominated diphenyl ether (PBDE) congeners and the risk of psoriasis. **(A)** PBB153; **(B)** PBDE28; **(C)** PBDE47; **(D)** PBDE85; **(E)** PBDE99; **(F)** PBDE100; **(G)** PBDE153; **(H)** PBDE154; **(I)** PBDE209. ln, natural logarithmic transformation; OR, odds ratio; CI, confidence interval.

RCS analyses revealed a significant positive dose–response relationship for PBDE153 (P-nonlinear = 0.881, P-overall = 0.012) (Figure 5G). Additionally, PBDE209 exhibited rising ORs with higher concentrations (Figure 5I), whereas PBDE85, PBDE99, and PBDE154 showed declining trends (Figures 5D,E,H). PBB153 displayed an S-shaped concentration-risk pattern (Figure 5A), while PBDE28, PBDE47, and PBDE100 presented an inverted U-shaped relationship (Figures 5B,C,F). However, these associations did not reach statistical significance.

To ensure the robustness of our findings against false positives from multiple comparisons, we applied FDR correction to all statistical tests for the nine individual BFRs. The resulting *q*-values are detailed in Supplementary Table S2 and summarized here. PBDE153 consistently showed the strongest associations: it remained statistically significant in the univariate model (*q* = 0.009) and for the linear trend across quartiles (*q* = 0.0315). Although the *q*-values for its multivariate association (*q* = 0.090) and its overall dose–response relationship in the RCS analysis (*q* = 0.108) exceeded the 0.05 threshold, the associations for PBDE153 remained the most consistent signal across all analytical frameworks. In contrast, all associations for the remaining eight BFRs were non-significant after FDR correction (all *q* > 0.26). Taken together, after comprehensive adjustment for multiple testing, PBDE153 emerged as the most prominent and consistent signal, warranting its selection for focused examination in subsequent mixture and mechanistic analyses.

### 3.3 Combined effects of BFRs on psoriasis

**Figure 6:**
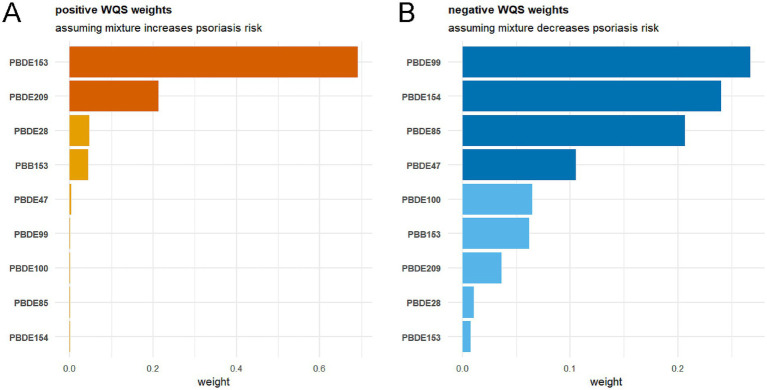
Association between the BFRs mixture and psoriasis risk in the WQS regression. **(A)** Positive correlation analysis of BFRs with psoriasis risk; **(B)** Negative correlation analysis of BFRs with psoriasis risk. The longest spline represents the BFR with the highest weight.

The cumulative relationship between multiple exposures to BFRs and psoriasis was evaluated using the WQS model (Figure 6). In the positive direction analysis (Figure 6A) (assuming the mixture increases psoriasis risk), we found a statistically significant association (OR = 1.709, 95% CI: 1.241–2.370, *p* = 0.001). PBDE153 emerged as the primary contributor to the WQS index (weight = 0.691), followed by PBDE209 (weight = 0.212). Conversely, in the negative direction analysis (Figure 6B) (assuming the mixture decreases risk), no significant association was observed (OR = 0.982, 95% CI: 0.765–1.255, *p* = 0.884), with PBDE99 (weight = 0.267), PBDE154 (weight = 0.240), and PBDE85 (weight = 0.207) as the main contributors.

**Figure 7:**
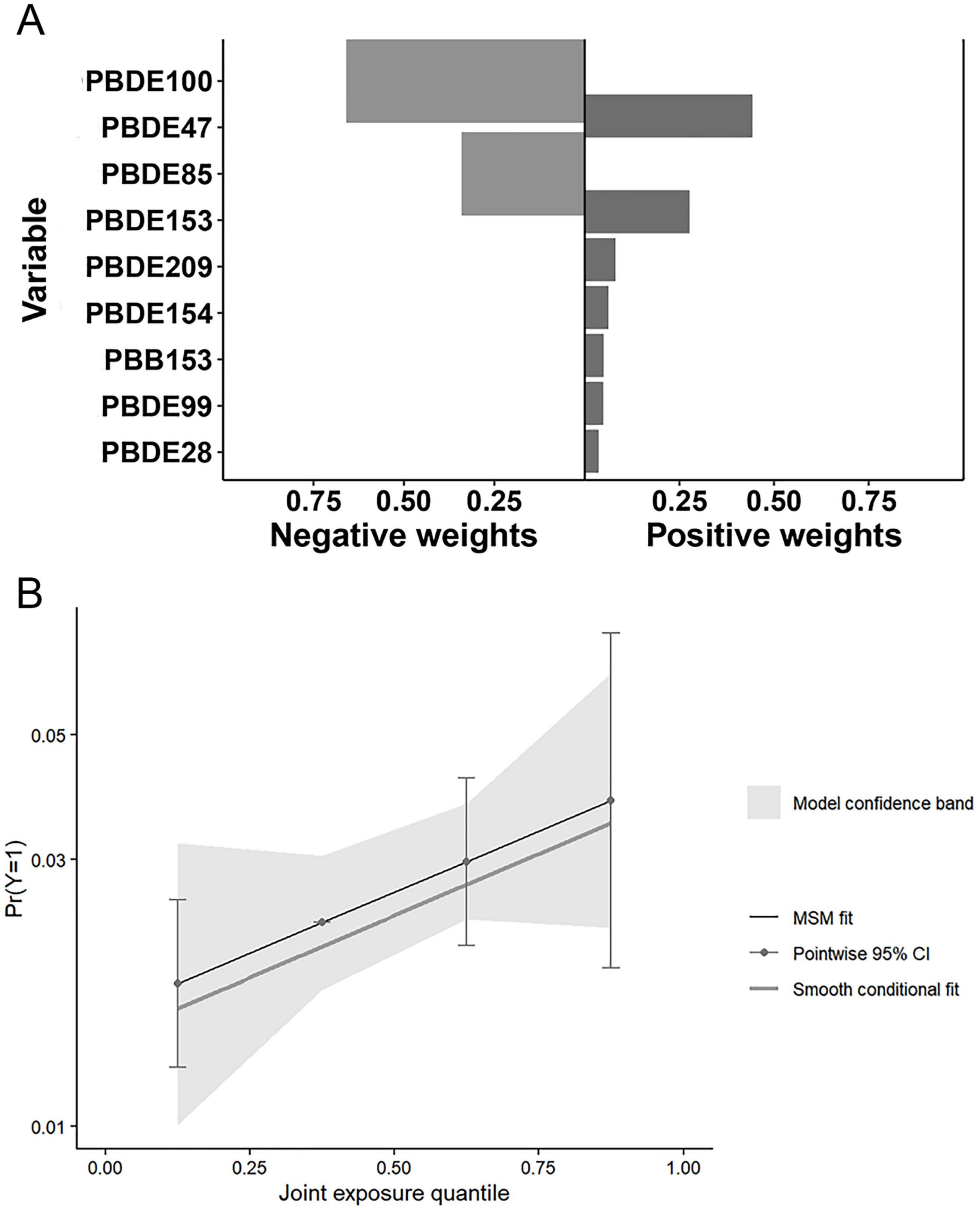
Association between the BFRs mixture and psoriasis risk in the QGC analysis. **(A)** Proportion of ln-transformed concentrations of BFRs in the QGC model; The size of each bar reflects its relative magnitude compared to other effects in the same direction. The depth of the bar graph corresponds to the overall effect size, with the darker side indicating the direction of the overall effect. **(B)** Combined effects of ln-transformed concentrations of BFRs in the QGC model. Gender, age, race, education level, PIR, marital status, BMI, alcohol intake, hypertension, diabetes, hypercholesterolemia, SII, SIRI and serum cotinine as adjusting variables for the model. PIR, the family income to poverty ratio; BMI, body mass index; SII, systemic immune-inflammation index; SIRI, systemic inflammation response index.

Quantile G-computation (Qgcomp) analysis (Figure 7) revealed a positive but non-significant association between the BFR mixture and psoriasis (psi1 = 0.273, 95% CI: −0.043 to 0.589, *p* = 0.090) (Figure 7B). PBDE47 (weight = 0.443) and PBDE153 (weight = 0.277) were identified as the primary positive contributors, while PBDE100 (weight = 0.659) and PBDE85 (weight = 0.341) showed negative associations (Figure 7A).

### 3.4 Sensitivity analysis with LASSO regression

To mitigate the impact of multicollinearity and identify key predictors, we employed LASSO regression. The cross-validation curve (Supplementary Figure S1) indicated the optimal lambda values for model selection. The lambda.min model retained a subset of variables, including the systemic inflammation marker SIRI (coefficient = 0.053), age (coefficient = 0.134), marital status (coefficient = 0.116), and four specific brominated compounds: PBDE209 (coefficient = 0.331), PBDE153 (coefficient = 0.247), PBDE85 (coefficient = −0.114), and PBDE154 (coefficient = −0.069). In contrast, the more stringent lambda.1se model retained only the intercept, indicating that no single variable had a strong enough independent effect to surpass the high penalty threshold when all others were shrunk to zero (Supplementary Table S3). This underscores the challenge of disentangling individual effects in highly correlated exposure mixtures. The variable importance plot derived from the lambda.min model (Supplementary Figure S2) visually emphasizes the relative contribution of these selected predictors.

### 3.5 Sensitivity analysis using Cook’s distance

To assess the potential influence of outliers on our primary findings, we conducted a diagnostic analysis using Cook’s distance for the model containing PBDE153 (Supplementary Table S4). The maximum Cook’s distance was 0.0226, with a median of 4.89 × 10^–6^, indicating generally low influence of individual observations on the regression model. Distribution analysis revealed that 99.58% of observations (*n* = 4,233) had Cook’s distances below 0.01, while only 0.42% (*n* = 18) fell between 0.01 and 0.05. No observations exceeded the conservative threshold of 1.0, which is commonly used to identify influential points in epidemiological studies. This diagnostic check indicates that our primary results are robust and not unduly influenced by any single outlier.

### 3.6 Subgroup interactions

**Figure 8:**
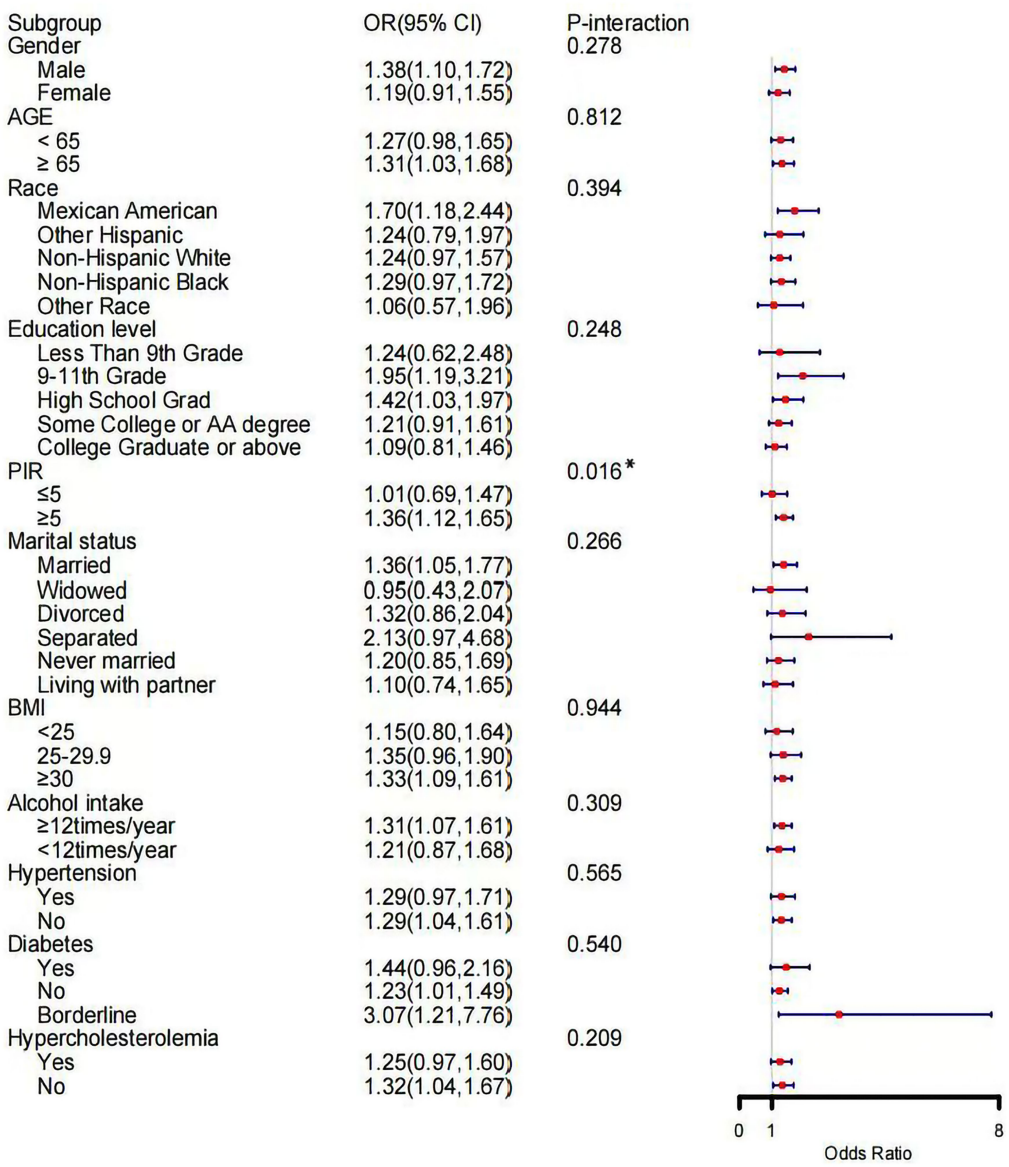
Subgroup analysis of the association between PBDE153 and the risk of psoriasis. PIR, the family income to poverty ratio; BMI, body mass index; OR, odd ratio; CI, confidence interval. ^*^p < 0.05.

In Figure 8, PBDE153 was positively associated with psoriasis in the following demographic groups: males over 65 years, Mexican Americans, individuals with 9th–11th grade education or a high school diploma, those with a PIR ≥ 5, married individuals, those with a BMI ≥ 30, frequent alcohol consumers, and those without hypertension, without or with borderline diabetes, and without hypercholesterolemia. Notably, a significant interaction was observed between PIR level and PBDE153 exposure (P-interaction = 0.016), suggesting enhanced susceptibility to PBDE153-associated psoriasis risk among individuals with a PIR ≥ 5.

### 3.7 Mediation analysis

**Figure 9:**
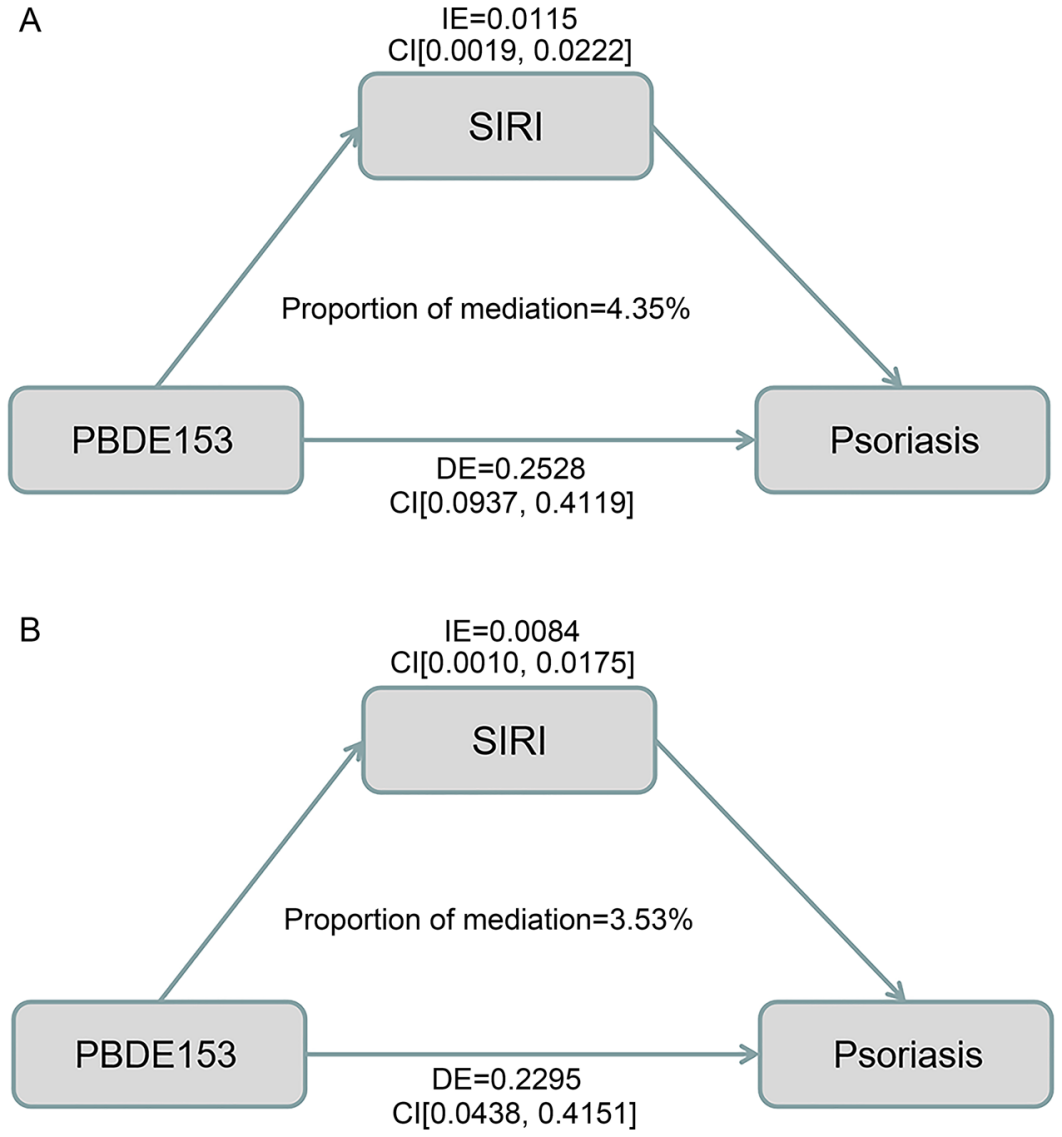
Mediating effect of inflammation and proportion mediated in the association between PBDE153 exposure and psoriasis prevalence. **(A)** Unadjusted model; **(B)** Adjusted for age and marital status. IE, indirect effect; DE, direct effect.

A parallel mediation analysis was conducted to investigate the potential mechanistic role of inflammatory markers in the PBDE153-psoriasis relationship (Figure 9). The SIRI was identified as a significant mediator, accounting for 4.35% of the total effect, with an indirect effect of 0.0115 (95% CI: 0.0019–0.0222) and a direct effect of 0.2528 (95% CI: 0.0937–0.4119) (Figure 9A). After adjusting for age and marital status (selected by LASSO regression), the mediation proportion was 3.53%, with an indirect effect of 0.0084 (95% CI: 0.0010–0.0175) and a direct effect of 0.2295 (95% CI: 0.0438–0.4151) (Figure 9B). Notably, the SII was not significantly associated with psoriasis in baseline characteristics (*p* = 0.251) and was therefore not included in the mediation analysis.

## 4 Discussion

Our comprehensive analysis employed multiple methodological approaches to evaluate the associations between individual and combined exposures to BFRs and psoriasis risk. The findings revealed a consistent and notable association between PBDE153 and psoriasis prevalence, which followed a positive dose–response pattern. Both WQS and Qgcomp analyses suggested a potential association between BFR mixtures and psoriasis, while the combined effects were not statistically significant in the latter. Notably, individuals with a higher PIR (≥5) showed greater susceptibility to PBDE153-associated psoriasis risk. The SIRI emerged as a critical mediator, accounting for 4.35% of the relationship between PBDE153 exposure and psoriasis risk (*p* < 0.05), highlighting inflammation as a potential mechanistic pathway. The association between PBDE153 and psoriasis remained statistically significant after FDR correction in both univariate analysis (*q* = 0.009) and for a linear dose–response trend (*q* = 0.0315). Although not meeting the strict significance threshold in multivariate and RCS models (*q* = 0.090 and 0.108, respectively), it consistently presented the strongest signal across all analyses, supporting a specific link. The attenuation of significance in the multivariate model is not uncommon and may be attributed to the adjustment for correlated covariates (e.g., other BFRs or inflammation markers) that may lie on the causal pathway or share common variance, thereby reducing the unique effect size attributed solely to PBDE153. The convergence of evidence from basic regression, trend tests, and complex dose–response modeling collectively suggests a link between PBDE153 exposure and psoriasis pathogenesis. However, these findings should be interpreted with caution. Firstly, the limited number of analyzed samples may have contributed to the lack of statistical power. Secondly, the high correlation coefficients (>0.70) and multicollinearity observed among most BFRs may have affected regression outcomes (29), potentially obscuring other meaningful associations. Thirdly, the varied risk curve patterns observed through restricted cubic spline (RCS) analysis suggest intricate dose–response relationships. This complexity may lead to mutual cancellation of effects during combined analyses, potentially resulting in non-significant findings. In addition, the small mediation proportion suggests that other pathways may also play important roles in the association between PBDE153 and psoriasis.

Our findings were further corroborated by a sensitivity analysis using LASSO regression, which was performed to address multicollinearity. The LASSO model consistently identified the inflammatory marker SIRI as a key predictor, reinforcing the role of inflammation in psoriasis. It also selected a subset of BFRs (PBDE209 and PBDE153 as positive correlates, and PBDE85 as a negative correlate) from the highly correlated mixture. The failure of no single BFR to survive the most stringent LASSO penalty (lambda.1se) highlights the intrinsic challenge of attributing risk to individual compounds within a tightly correlated mixture and underscores the utility of employing multiple complementary statistical approaches. Diagnostic checks using Cook’s distance also strengthened the validity of our findings. The absence of influential observations (Cook’s distance > 1) indicates that our results are not disproportionately influenced by any single data point, enhancing confidence in the observed association between PBDE153 and psoriasis risk.

The biological plausibility of our findings aligns with current understanding of psoriasis pathogenesis. This chronic inflammatory condition involves complex interactions between immune cells and keratinocytes, characterized by the activation of T helper 17 cells and subsequent IL-17 production, which leads to keratinocyte hyperproliferation and plaque formation (30). Keratinocytes further amplify the inflammatory cascade by releasing pro-inflammatory mediators, thereby establishing a self-perpetuating cycle of inflammation (31). Although direct mechanistic studies on PBDE153 in psoriasis are currently lacking, research in related contexts provides compelling parallels. Previous experimental studies suggest that PBDEs may enhance the production of pro-inflammatory cytokines and intrude intrude into the pathogenesis of psoriasis. These compounds can enhance the production of TNF-α and IL-6 (32), promote Th17 differentiation, and induce oxidative stress and endoplasmic reticulum stress in keratinocytes, thereby exacerbating psoriatic inflammation (30, 33). Additionally, the bioaccumulation of BFRs raises concerns about their endocrine-disrupting effects which exacerbate inflammatory conditions. Specifically, by inducing thyroxine (T4)-metabolizing enzymes like UDPGT and through hydroxylated metabolites displacing T4 from transthyretin (TTR), they suppress T4 levels. Since hypothyroidism is a potential pro-inflammatory state, this thyroid axis disruption may indirectly foster a systemic inflammatory environment (34, 35). The shared involvement of inflammatory pathways formed the rationale for exploring inflammation as a potential mediator in the observed association of PBDE153 and psoriasis.

Recent advances have established SII and SIRI as valuable inflammatory biomarkers, widely utilized in investigating the relationship between chronic inflammation and diseases such as cancer and metabolic disorders (36). These indices have also shown positive correlations with psoriasis risk (22). Our baseline analysis revealed significant differences between-group in SIRI, PBDE153 levels, and age, prompting a mediation analysis with PBDE153 as the independent variable, SIRI as the mediator, and psoriasis status as the outcome. The mediation analysis identified a statistically significant mediating role for SIRI in the relationship between PBDE153 and psoriasis. In the unadjusted model, SIRI accounted for 4.35% of the total effect (indirect effect = 0.0115, 95% CI: 0.0019–0.0222; direct effect = 0.2528, 95% CI: 0.0937–0.4119). After adjusting for the LASSO-selected covariates (age and marital status), the mediation proportion remained significant at 3.53% (indirect effect = 0.0084, 95% CI: 0.0010–0.0175; direct effect = 0.2295, 95% CI: 0.0438–0.4151). This finding contributes to our understanding of a potential pathway linking BFRs to psoriasis risk and may inform future preventive strategies. However, the modest mediation proportion strongly implies that other mechanisms, such as direct cellular toxicity and neurotoxicity, are likely involved and warrant investigation.

The results identified a statistically significant mediating role for SIRI in the relationship between PBDE153 and psoriasis, enhancing our understanding of the mechanism linking BFRs to psoriasis risk and potentially informing preventive strategies.

The significant association between PBDE153 and psoriasis is consistent with emerging evidence that links this compound to other health conditions through similar pathogenic mechanisms. For instance, elevated serum PBDE153 and PBDE100 concentrations have been independently associated with an increased risk of hypertension, potentially mediated by inflammatory responses and oxidative stress (37). Similarly, research has identified PBDE-153, PBDE-209, and PBB-153 as significant negative predictors of bone mineral density, possibly due to their disruption of endocrine and metabolic processes (38).

Our stratified analysis revealed a notable interaction between socioeconomic status (measured by PIR) and PBDE153-associated psoriasis risk. Several factors may explain this relationship. Higher-income households often exhibit increased exposure to environmental pollutants due to greater consumption of flame retardant-containing products and more frequent replacement of consumer goods (39). Indoor environments in affluent households may particularly concentrate PBDE exposure, as research has linked PBDE-containing furniture to elevated levels of these compounds in household dust and, consequently, in human serum (40). These findings suggest several practical interventions to mitigate the impact of PBDEs on psoriasis risk: minimizing the use of products containing PBDEs, particularly older electronics and furniture, reducing the frequency of consumer goods replacement, and implementing regular cleaning practices to decrease dust accumulation (41, 42).

This investigation presents several notable strengths. Primarily, it is the first systematic examination of the relationship between psoriasis risk and BFR exposure. Additionally, our analysis leverages data from a large, population-based database that adheres to robust quality control protocols, thereby enhancing the reliability of our findings. Nevertheless, several limitations warrant consideration. Firstly, despite the relatively large size of the research sample, the number of psoriasis cases (*n* = 123) remains comparatively limited. In the context of subgroup and interaction analyses, a small number of outcome events can introduce substantial variability in the estimation of effect sizes. This variability may be disproportionately affected by a few events or may fail to detect actual differences due to inadequate statistical power. Therefore, the findings from subgroup analyses should be interpreted with caution and require further validation. Secondly, while the prevalence of psoriasis observed in this study (2.9%) closely aligns with the global incidence rate of 2–3% reported in previous research (15), it is important to note that the data on psoriasis were obtained from self-reports, which may be susceptible to reporting bias. This susceptibility can lead to misclassification bias regarding psoriasis status, potentially resulting in inaccurate estimations of the associations between exposure to BFRs and psoriasis. Thirdly, although liquid–liquid extraction is a common method for measuring BFRs, it often suffers from matrix effects. These effects occur when other substances in the sample interfere with the analysis, potentially leading to inaccurate readings of BFR levels. Fourthly, single time-point measurements of BFR levels may not accurately represent long-term exposure patterns, especially for substances whose levels fluctuate due to environmental factors. Additionally, the analysis was limited to nine BFRs, potentially overlooking other relevant compounds. While our diagnostic checks using Cook’s distance revealed no influential observations that would alter our conclusions, we acknowledge that other unmeasured confounding factors might still influence the observed associations. Finally, due to the cross-sectional nature of the study, the cross-sectional design precludes causal inference. To address these limitations and build upon our findings, we recommend specific future studies. First, prospective longitudinal cohorts with repeated measurements of BFRs are essential to establish temporal sequence and reduce exposure misclassification. Second, targeted mechanistic investigations are warranted. These could include *in vitro* models using human keratinocytes or immune cells to examine the direct effects of PBDE153 on the IL-23/Th17 axis, a central pathway in psoriasis. Additionally, animal models of psoriasis could be employed to assess if pre-exposure to PBDE153 exacerbates disease severity. Such studies are crucial to confirm the association and elucidate the underlying biological pathways.

## 5 Conclusion

Our findings suggest a positive association between PBDE153 exposure and psoriasis prevalence, with inflammation identified as a potential mediating pathway. Future prospective longitudinal studies and targeted experimental research are necessary to validate this association and clarify the underlying mechanisms.

## Data Availability

Publicly available datasets were analyzed in this study. This data can be found at: https://wwwn.cdc.gov/nchs/nhanes/Default.aspx.
