# Supplementary material for: Inflammation mediation of the association between brominated flame retardants and psoriasis among U.S. adults
Source: Front Public Health. 2025 Dec 10;13:1602943. doi: 10.3389/fpubh.2025.1602943 (PMC12727945; doi:10.3389/fpubh.2025.1602943)
Supplement: Supplementary file 1 [file Supplementary_file_1.docx]

Supplementary Table S1 Analytical characteristics of brominated flame retardants (BFRs) in the study population (NHANES 2005-2006, 2009-2014)

| Compound (Abbreviation) | Full Chemical Name | % > LOD | Samples< LOD (n) |
| --- | --- | --- | --- |
| PBDE28 | 2,4,4′-Tribromodiphenyl ether | 1 | 0 |
| PBDE47 | 2,2',4,4'-Tetrabromodiphenyl ether | 98.2 | 20 |
| PBDE85 | 2,2',3,4,4'-Tetrabromodiphenyl ether | 1 | 0 |
| PBDE99 | 2,2',4,4',5-Pentabromodiphenyl ether | 87.0 | 141 |
| PBDE100 | 2,2',4,4',6-Pentabromodiphenyl ether | 1 | 0 |
| PBDE153 | 2,2',4,4',5,5'-Hexabromodiphenyl ether | 1 | 0 |
| PBDE154 | 2,2',4,4',5,6'-Hexabromodiphenyl ether | 90.0 | 108 |
| PBDE209 | Decabromodiphenyl ether | 84.6 | 167 |
| PBB153 | 2,2',4,4',5,5'-Hexabromobiphenyl | 78.1 | 236 |

Note: LOD = limit of detection. Values below LOD were imputed as LOD/√2 for statistical analyses.

Supplementary Table S2 P-values and FDR-adjusted Q-values for associations between individual BFRs and psoriasis across different statistical models

| BFR Congener | P (Univariate) | Q (Univariate) | P (Multivariate) | Q (Multivariate) | P (Trend) | Q (Trend) | P (RCS) | Q (RCS) |
| --- | --- | --- | --- | --- | --- | --- | --- | --- |
| PBB153 | 0.963 | 0.963 | 0.652 | 0.734 | 0.987 | 0.987 | 0.359 | 0.765 |
| PBDE28 | 0.962 | 0.963 | 0.791 | 0.791 | 0.565 | 0.636 | 0.783 | 0.881 |
| PBDE47 | 0.338 | 0.435 | 0.462 | 0.594 | 0.412 | 0.53 | 0.73 | 0.881 |
| PBDE85 | 0.098 | 0.176 | 0.186 | 0.335 | 0.179 | 0.392 | 0.425 | 0.765 |
| PBDE99 | 0.181 | 0.271 | 0.317 | 0.476 | 0.181 | 0.392 | 0.583 | 0.874 |
| PBDE100 | 0.065 | 0.176 | 0.119 | 0.286 | 0.261 | 0.392 | 0.247 | 0.765 |
| PBDE153 | 0.001 | **0.009** | 0.01 | 0.09 | 0.007 | **0.032** | 0.012 | 0.108 |
| PBDE154 | 0.058 | 0.176 | 0.127 | 0.286 | 0.245 | 0.392 | 0.954 | 0.954 |
| PBDE209 | 0.085 | 0.176 | 0.059 | 0.265 | 0.001 | **0.009** | 0.293 | 0.765 |

Abbreviations: BFR, brominated flame retardant; RCS, restricted cubic spline; FDR, false discovery rate (Benjamini-Hochberg procedure). Values in bold indicate statistical significance after FDR correction (q < 0.05)

Supplementary Table S3 Coefficients of variables selected in the LASSO regression models (lambda. min and lambda.1se)

| Variable | Coefficient (lambda. min) | Coefficient (lambda. 1se) |
| --- | --- | --- |
| (Intercept) | -4.860 | -3.513 |
| AGE | 0.134 | 0 |
| DMDMARTL | 0.116 | 0 |
| SIRI | 0.053 | 0 |
| PBDE85 | -0.114 | 0 |
| PBDE153 | 0.247 | 0 |
| PBDE154 | -0.069 | 0 |
| PBDE209 | 0.331 | 0 |

Supplementary Table S4 Distribution of Cook's distance and sensitivity analysis results for the association between PBDE153 and psoriasis

| Cook's distance range | Number of observations |
| --- | --- |
| 0-0.01 | 4233 |
| 0.01-0.05 | 18 |
| 0.05-0.1 | 0 |
| 0.1-0.5 | 0 |
| 0.5-1.0 | 0 |
| >1.0 | 0 |

Maximum Cook's distance: 0.0226

Threshold for influential observations: 1.0

Number of influential observations excluded: 0
