# Supplementary figures and images for: Inflammation mediation of the association between brominated flame retardants and psoriasis among U.S. adults
Source: Front Public Health. 2025 Dec 10;13:1602943. doi: 10.3389/fpubh.2025.1602943 (PMC12727945; doi:10.3389/fpubh.2025.1602943)

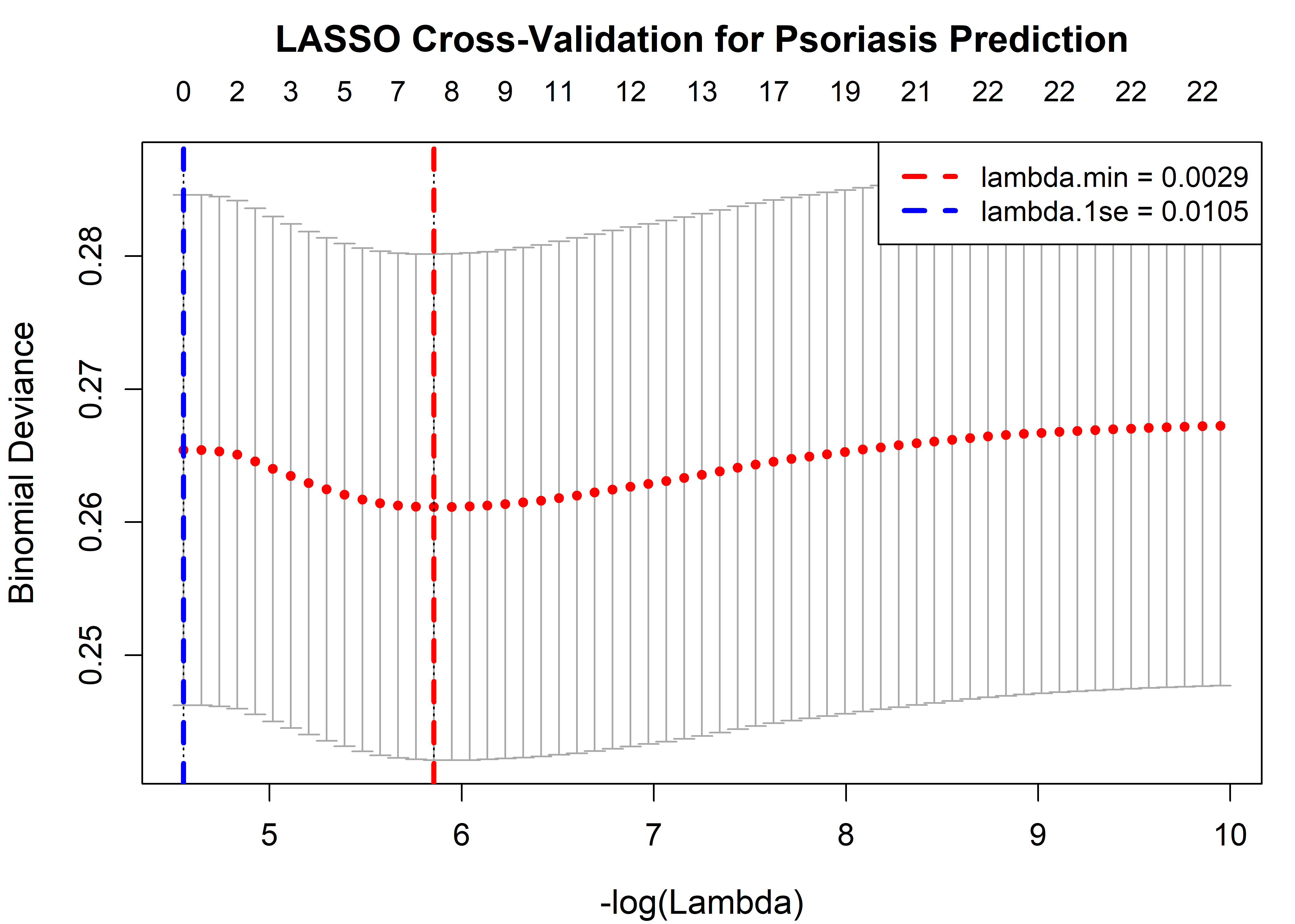

Supplement: Supplementary file 2 [file Image_1.tif]

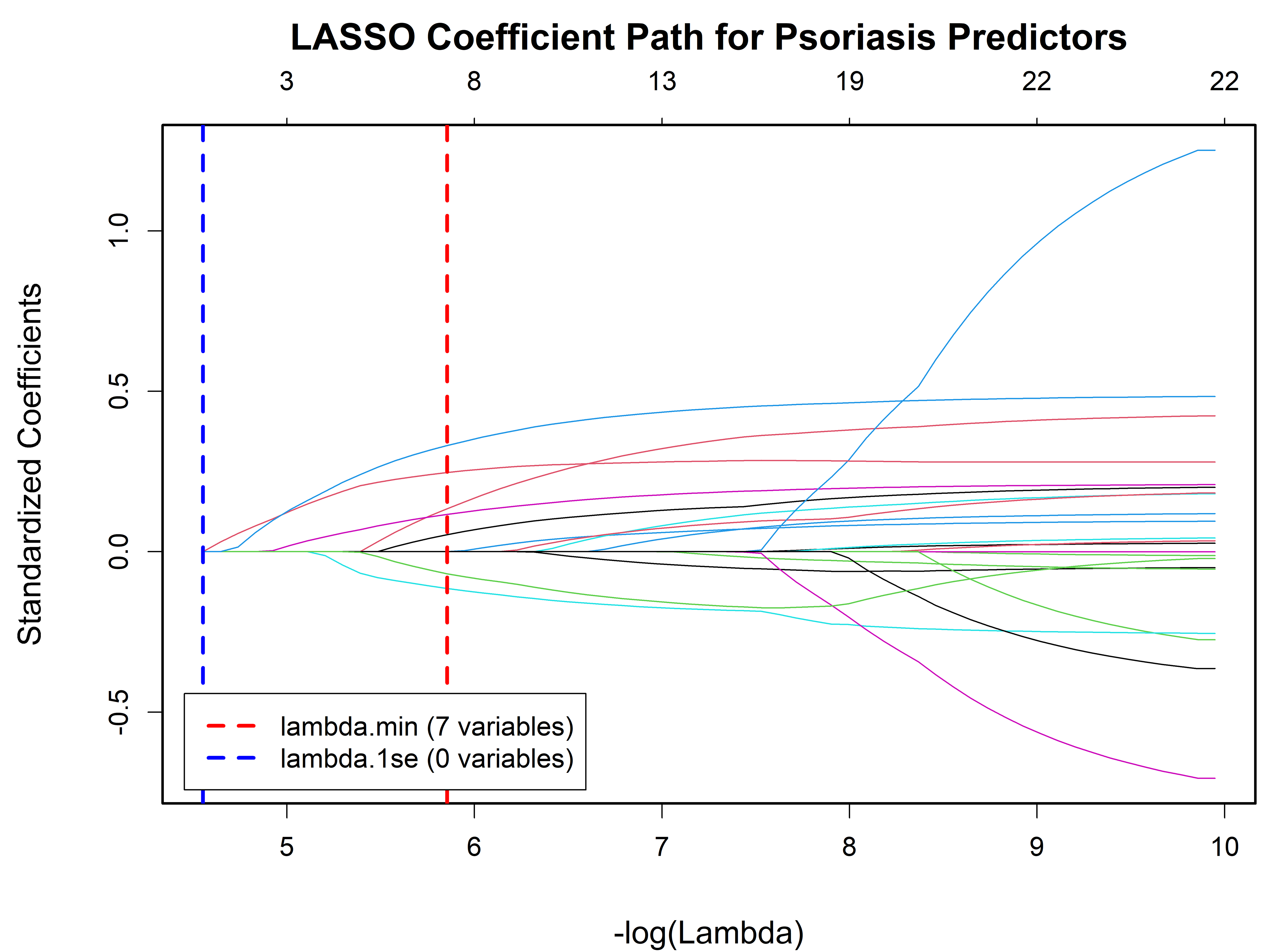

Supplement: Supplementary file 3 [file Image_2.tif]
